# Supplementary material for: Integrative molecular analyses of lineage identity and morphology in aggressive variant prostate cancer
Source: NPJ Precis Oncol. 2026 Apr 9;10:208. doi: 10.1038/s41698-026-01400-6 (PMC13247060; doi:10.1038/s41698-026-01400-6)
Supplement: Supplementary file 1 — supptext [file 41698_2026_1400_MOESM1_ESM.pdf]

Supplementary Information

Integrative molecular analyses of lineage identity and morphology in aggressive variant prostate cancer

Chennan Li *et al.*

- Supplementary Table
- Supplementary Figure

Supplementary Table 1

Sample	Origin	Small cell features	Location
CP02107	transformed	positive	prostate
CP06649	transformed	negative	adrenal mass
CP08724	transformed	positive	inguinal mass
CP11759	transformed	negative	rectal mass
CP12999	transformed	negative	prostate
CP13370	de novo	positive	prostate
CP14320	transformed	positive	liver
CP14377	de novo	positive	liver
CP14866	transformed	positive	liver
CP15493	de novo	positive	prostate
CP15668	transformed	positive	liver
CP17102	de novo	positive	prostate

Supplementary Table 1. Biopsy location for each AVPC specimen.

Supplementary Figure 1

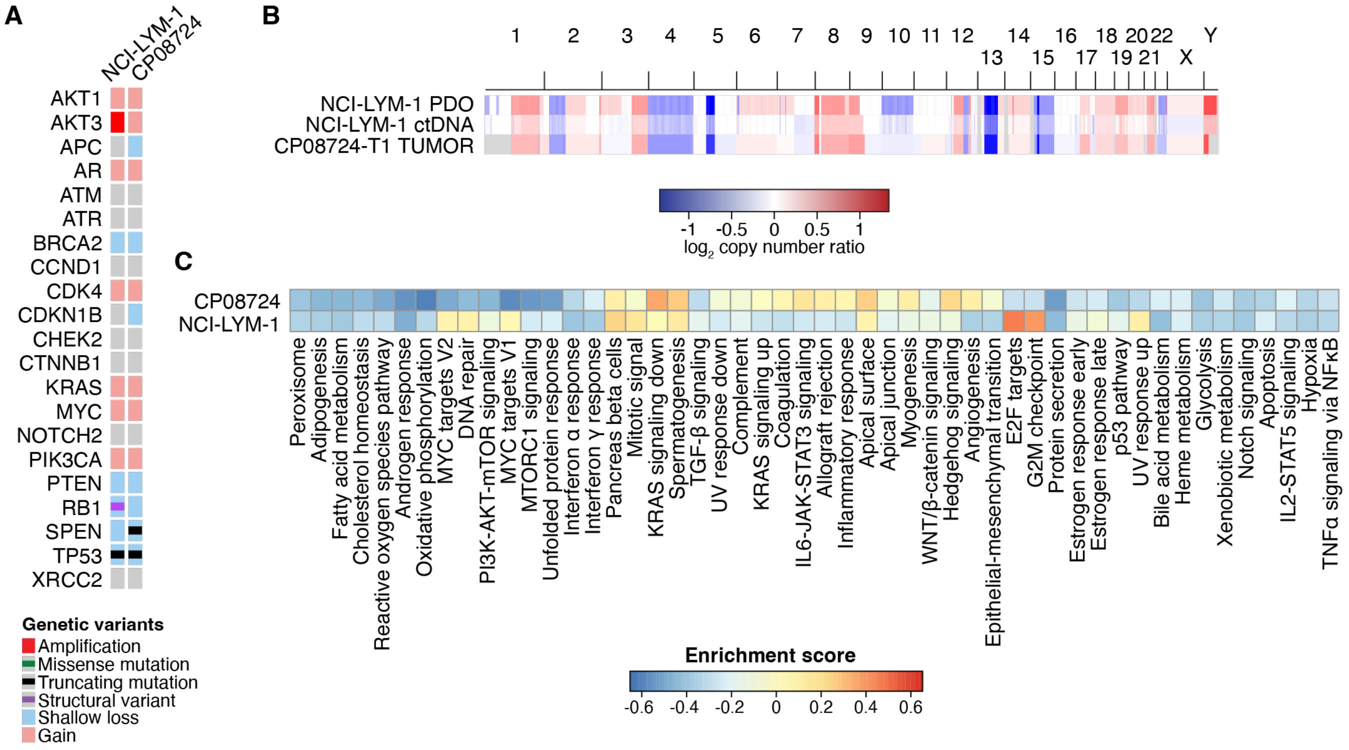

Supplementary Figure 1. Comparison of molecular features between NCI-LYM-1 and donor tumor biopsy. (A) Oncoprint depicting copy number and mutational status of selected prostate cancer genes in NCI-LYM-1 and donor tumor biopsy. All mutations shown were curated for known oncogenic status. **(B)** Whole-genome somatic copy-number estimates derived from whole-genome sequencing NCI-LYM-1 and donor circulating tumor DNA (ctDNA), and whole-exome sequencing of donor tumor biopsy. **C)** Heatmap depicting unsupervised nonparametric gene set variation analysis for NCI-LYM-1 and donor tumor biopsy transcriptomes projected against the mSigDB Hallmarks gene sets.
